# Dendritic spine degeneration is associated with age-related decline in recognition and spatial memory in male mice

**DOI:** 10.1007/s00429-025-03002-7

**Published:** 2025-08-28

**Authors:** Elibeth Monroy, Leonardo Aguilar-Hernandez, Fidel de la Cruz-López, Gonzalo Flores, Julio César Morales-Medina

**Affiliations:** 1 Lab. Neuropsiquiatría, Instituto de Fisiología, Benemérita Universidad Autónoma de Puebla, Puebla, 72570 México; 2 Escuela Nacional de Ciencias Biológicas, Instituto Politécnico Nacional, Mexico city, Mexico; 3 Centro de Investigación y de Estudios Avanzados del Instituto Politécnico Nacional, Unidad Tlaxcala, Ixtacuixtla, Tlaxcala 90000 México

**Keywords:** Aging, CA1 hippocampus, Morris water maze, Object recognition, Prefrontal cortex

## Abstract

Human populations are experiencing an increase in aging, which is associated with cognitive deficits. Animal models of aging have shown that these behavioral impairments are associated with neuroarchitecture modifications in the prefrontal cortex (PFC) and hippocampus; however, most studies have focused on rats or lack multiple key ages. In this study, we evaluated spatial and recognition memory in male mice at critical ages [3 months (M), 6, 12 and 18] using the Morris water maze (MWM) and novel object recognition test (NORT), respectively. Moreover, we quantified dendritic arborization, spine density and the type of spines in the PFC, CA1 hippocampus and nucleus Accumbens Core (NAcC). Locomotion, assessed in the first phase of NORT, revealed age-dependent reductions. Notably, the 18 M group revealed significant recognition memory deficits. Spatial memory impairments were especially evident at the 12 M group in the MWM. Spine density was increased at 6 M in the NAcC, whereas a reduction was noted at 12 M and 18 M in the PFC. Morphological assessment of spines indicated age-dependent changes, including a notable increase in the proportion of thin spines in the CA1 and PFC regions. However, dendritic arborization remained largely unchanged across the examined brain regions and age groups. Overall, our findings observed age-dependent alterations in memory and morphological alterations in spines in mice, emerging as possible contributors to cognitive decline. These results highlight the potential for anti-aging interventions targeting synaptic structures to enhance cognitive health and extend the healthspan of aging individuals.

**Supplementary Information:**

The online version contains supplementary material available at 10.1007/s00429-025-03002-7.

## Introduction

Aging is characterized by a progressive and irreversible physical and functional decline (Benoit et al. 2011). Its etiology is associated with the gradual accumulation of damage and cellular waste (da Silva and Schumacher 2021). As a consequence, a sustained state of oxidative stress (Liguori et al. 2018; Hajam et al. 2022), inflammation (Borgoni et al. 2021; Walker et al. 2022), and cellular senescence (Calcinotto et al. 2019) is established. Due to its high metabolic rate, the brain is one of the organs that is most susceptible to aging. Reaching this stage of life is the main risk factor for neurodegenerative disorders such as Alzheimer’s and Parkinson’s diseases (Hou et al. 2019; Salthouse 2019). However, even in normal aging there are declines in cognitive functions such as memory and learning (Uryash et al. 2020; Hendrickx et al. 2022; Yang et al. 2023). The intensity of these cognitive declines and the age of the individual has been correlated (Nyberg et al. 2012; Pliatsikas et al. 2019; Spreng and Turner 2019). Memory decline has been observed in aged rodents and even in rodents with induced aging, findings that are associated with an increase in oxidative stress and inflammation (Rosenzweig and Barnes 2003; Li et al. 2022; Rahman et al. 2022; Aguilar-Hernandez et al. 2023). Episodic memory is one of the most affected during aging as observed in animal models (Sanches et al. 2022).

Cognitive functions arise from the functional interaction between neurons of the limbic-cortical system such as the prefrontal cortex (PFC), hippocampus and nucleus Accumbens (NA) (Brown and Sharp 1995; O’Neill et al. 2013; Spellman et al. 2015; Sekeres et al. 2018; Yavas et al. 2019; Rolls 2022). Although cumulative evidence supports the involvement of the dorsal hippocampus in spatial learning, additional brain regions also contribute to this process. The NA, which receives projections from the hippocampus, is considered a key site for the integration of spatial and reward-related information (Brown and Sharp 1995; Braun et al. 2016). Lesions to the NA have been shown to impair performance in the Morris water maze (MWM). Furthermore, the PFC is implicated in both egocentric and allocentric navigation, and damage to this region results in deficits in tasks such as the radial arm maze (Kolb et al. 1994; Braun et al. 2016). Activity-dependent plasticity is a central mechanism in memory formation (Menard et al. 2015). These events occur through chemical synapses mediated primarily by glutamate on receptor structures called dendritic spines (Mattison et al. 2014; Pinzon-Parra et al. 2022). Dendritic spines are protrusions from the dendritic surface that make contact with axon terminals (von Bohlen Und Halbach 2023). While learning processes are associated with the formation of new dendritic spines, memory is related with the temporary maintenance of these structures in their most efficient forms (Luine et al. 2018; Bencsik et al. 2019; Chidambaram et al. 2019). A correlation between alterations in the density and morphology ​​of dendritic spines with the performance of cognitive functions has been propossed (Liu et al. 2018).

In aged animals with impaired cognition, neuronal atrophy and reduced dendritic spine density are observed in regions involved in cognitive processes such as the CA1 hippocampus and PFC (Freire-Cobo et al. 2023). These regions show major dendrite and spines modifications due to aging changes (Bartsch and Wulff 2015). A previous study from our group have shown reductions in dendritic spine density and changes in the proportion of dendritic spine types, as well as declines in recognition memory in male rats (Aguilar-Hernandez et al. 2020). In the present study, we evaluated dendritic length and complexity, along with the density and morphology of dendritic spines in mice of different ages within the CA1 region of the hippocampus, PFC, and the nucleus Accumbens Core (NAcC), to assess the impact of aging and its relationship with recognition and spatial memory. To capture distinct stages across the lifespan, we included 3-month-old (3 M) mice representing early adulthood, 6 M mice corresponding to early middle age, 12 M mice reflecting midlife, and 18 M mice indicative of the aging stage, as defined by Dutta and Sengupta (2016).

## Methodology

### Animals

**Fig. 1:**
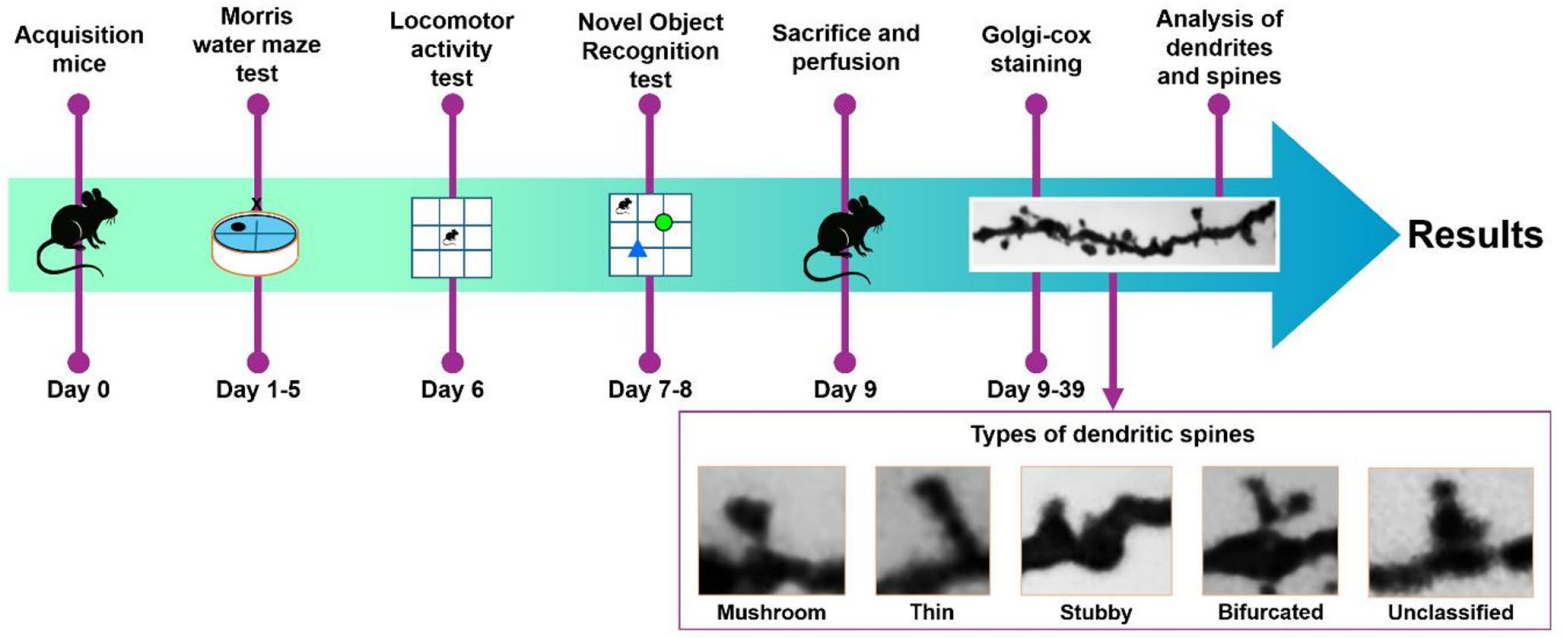
Timeline methodology. Following mice acquisition, the Morris water maze was performed during 5 days. Locomotor activity was assessed 24 h later. One day later, the Novel Object Recognition test was performed for 2 days. The following day, mice were sacrificed, and their brains were collected and maintained in Golgi-cox solution for 30 days. After that, brains were sectioned and mounted on slides for their observation. Dendrites and dendritic spines morphology were analyzed

C57BL6 mice aged 3, 6, 12 and 18 months (*n* = 10 per group) were obtained from the Claude Bernard bioterium of the Benemérita Universidad Autónoma de Puebla (BUAP, Puebla, Mexico). The mice were maintained under constant temperature and humidity conditions (24 °C) and water and food *ad libitum*. All procedures complied with the National Institutes of Health Guide for the Care and Use of Laboratory Animals, the technical guidelines for animals in the laboratory issued by SAGARPA Mexico (NOM-062 ZOO-1999), the BUAP animal care committee and ARRIVE guidelines (Kilkenny et al. 2010). During experimentation, any animals showing signs of distress were assessed by a veterinarian and, if necessary, euthanized to minimize animal suffering. The complete chronology of the methodology is presented in the Fig. 1.

### Behavior analysis

A battery of behavioral tests was performed. The MWM was assessed first followed by the novel object recognition test (NORT).

#### Spatial memory analysis

At the stablished ages, the MWM was used to evaluate spatial memory as described previously (Morales-Medina et al. 2013; Gonzalez-Granillo et al. 2022). Each mouse was placed in a pool filled with white water dyed with titanium dioxide (0.01%) which had a 3 cm platform hidden 2 cm below the water level. The animals repeated the test starting from different places in each trial, having to locate the platform to protect themselves on it. The pool had signs in each quadrant so that the rodent could locate the quadrant where the platform was located. The animals underwent three trials a day for 5 consecutive days. Each trial had a maximum duration of 90 s. If the rodent did not locate the platform, it was manually placed on it. Once on the platform, the rodent remained on it for 15 s before being removed and each trial concluded. At the end, the rodents were dried and placed under heat lamps to normalize their temperature. In each test, the time it took the rodent to locate the platform and the time it remained in the quadrant where it was located were quantified.

#### Recognition memory analysis

After the MWM test, the rodent was placed in a 40 cm cube box on each side for 10 min for habituation (Lima-Castaneda et al. 2023; Espinoza et al. 2025). The distance traveled in this test was quantified to obtain locomotor activity in a novel environment. 24 h later, the NORT was used to assess recognition memory using the same test box. The test consisted of three phases (Fig. 1); (a) Familiarization: Two identical objects (familiar objects) were placed in the box and the mouse was subsequently introduced for 6 min. (b) Short-term (ST) memory: 2 h after the familiarization phase. One of the familiar objects was replaced by a different object (novel object) and the rodent was again introduced for 6 min. (c) Long-term (LT) memory: 24 h after the ST phase. The novel object was replaced by a new novel object, then the same test was repeated with each mouse. Each trial was videotaped, and the time that the mouse spent exploring each of the objects was quantified. The discrimination index (DI) was then determined by obtaining the quotient of the time spent exploring the novel object compared to the total exploration time.

### Dendritic morphology

#### Obtaining and staining tissue

After the behavioral tests, the rodents were sacrificed using an overdose of sodium pentobarbital (60 mg/kg) (Morales-Medina et al. 2007; Flores et al. 2015). Once the rodent was completely anesthetized, cardiac perfusion was performed using saline solution, its brain was obtained and placed in Golgi-cox solution for 30 days. It was then placed in a 30% sucrose solution for 7 days and then sectioned with a vibratome (Leica systems) obtaining 200 μm thick sections. The sections were placed on slides previously treated with gelatin and the staining was developed using sodium hydroxide (%) for 30 min, the reaction was stopped using Kodak rapid fixative for 30 min and then the tissues were dehydrated with ethanol in increasing concentrations, finally they were cleared with xylene and mounted with synthetic resin.

#### Analysis of dendritic arborization

Fully impregnated and isolated neurons from the CA1 hippocampus, NAcC and PFC were analyzed, their localization was made according to the Mice Brain Atlas (Paxinos 2007). Specifically, the basilar arbor in pyramidal neurons within the anterodorsal CA1 subregion in the pyramidal stratum (Bregma − 1.46 to −2.54 mm) were included in this study (Silva-Gomez et al. 2003; Flores et al. 2005). For NAcC, medium spiny neurons located in the core region (Bregma 1.78 to 0.74 mm) were selected. For these neurons, all the dendrites were included in the analysis of dendritic arborization. In PFC, pyramidal neurons located in the layer 3, or pyramidal external layer, within the cingulate cortex (Bregma 2.34 to −0.1 mm) were selected. Of these cells, the basilar arbor was analyzed. A total of 10 neurons for each region were analyzed for each mouse.

For each animal, a trained observer, blinded to the experimental groups, used a camera lucida on a DMLS Leica microscope (400 ×) to bi-dimensionally reproduce five neurons from each hemisphere. Each neuron’s complete dendritic arbor was reconstructed in two dimensions and evaluated with Sholl analysis (Sholl 1953; Flores et al. 2015). A transparent grid of concentric rings spaced 5 mm apart was centered on the soma, and the number of ring intersections provided estimates of total dendritic length (TDL) and arbor complexity. Arborization was also quantified by counting every Y‑shaped bifurcation at successive branch orders.

#### Dendritic spine analysis

Dendritic spine density was performance selecting a fully impregnated and isolated neuron from each region as describe above. For each selected neuron, a distal and terminal dendrite, i.e., from 3rd order or beyond, considering the dendrites that emerge from the soma the 1 st order ones, were analyzed. Dendrites analyzed were in the same dendritic arbor detailed above. Using a camera lucida on a DMLS Leica microscope (1000 ×), a distal dendritic of 30 μm segment was reconstructed (5.4 cm). Subsequently, the number of dendritic spines was count and the mean of dendritic spines in 10 μm was estimated (Aguilar-Hernandez et al. 2020).

Dendritic spine morphology was evaluated on the same dendritic segments previously selected for spine density analysis, following established protocols described by our group (Tendilla-Beltran et al. 2019a, b). Using a DMLS Leica microscope equipped with a 2× lens and a total magnification of 2000×, a total of 100 consecutive spines per neuron were analyzed. Spines were classified based on head and neck morphology into five categories: (1) mushroom spines, characterized by a large, prominent head and a well-defined neck; (2) thin spines, with elongated shape and similar head and neck diameters; (3) stubby spines, short and wide without a discernible neck; (4) bifurcated spines, displaying a single neck with two heads; and (5) unclassified spines, which did not conform to any of the defined categories. Examples of each type of dendritic spine is showed in the Fig. 1. All spine density and morphological classifications were conducted by a trained observer blinded to the experimental conditions.

### Statistical analysis

Results are presented as mean ± standard error of the mean (SEM), data were analyzed with a one-way ANOVA. GraphPad Prism version 8.0 was used, differences with a *P* < 0.05 were considered as significative and the Bonferroni test was used as a post-hoc analysis.

## Results

### Spatial and recognition memory is reduced in aged rodents

#### Locomotor activity

**Fig. 2:**
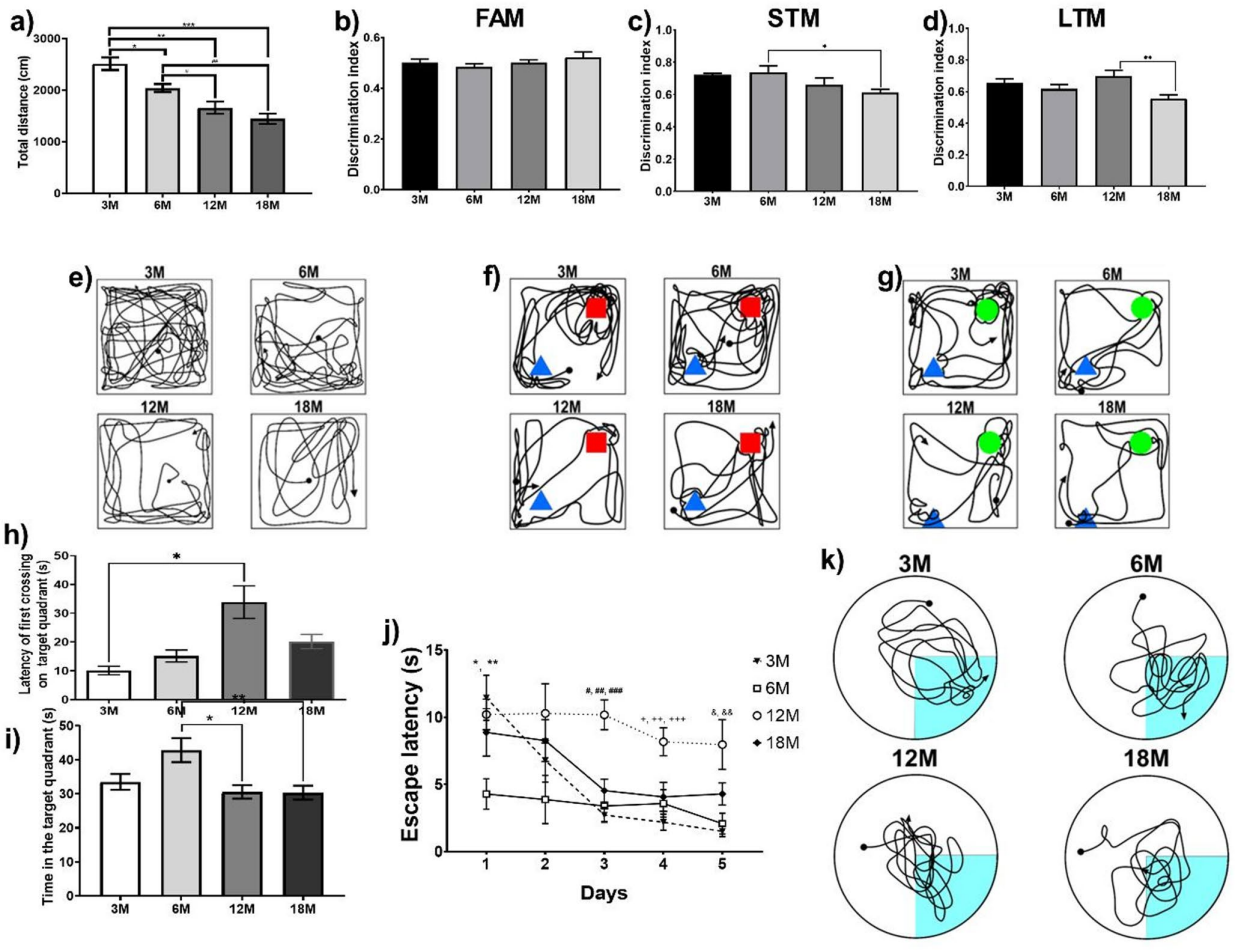
Spatial and recognition memory across age. **(a)** Mean of total distance traveled, significant differences were found between *3 M vs. 6 M (*P* = 0.0077, *n* = 10), **3 M vs. 12 M (*P* = 0.0067, *n* = 10), ***3 M vs. 18 M (*P* < 0.0001, *n* = 10), ^#^6 M vs. 12 M (*P* = 0.0357, *n* = 10), and ^##^6 M vs. 18 M groups (*P* = 0.0101, *n* = 10). **(b)** DI in the familiarization phase of the NORT, there are no significant differences. **(c)** DI in ST memory, significant differences were found between *6 M vs. 18 M groups (*P* = 0.0385, *n* = 8). **(d)** DI in LT memory, significant differences were found between **12 M vs. 18 M groups (*P* = 0.0107, *n* = 8). **(e)** Two-dimensional representation of the path of a representative rodent of each age. **(f)** Two-dimensional representation of the path in ST phase of a representative rodent of each age. **(g)** Two-dimensional representation of the path in LT phase of a representative rodent of each age. **(h)** Latency of first crossing on target quadrant, significant differences were found between *3 M vs. 12 M groups (*P* < 0.0001, *n* = 8). **(i)** Time spent in the target quadrant, significant differences were found between *6 M vs. 12 M (*P* = 0.0102, *n* = 8), and **6 M vs. 18 M groups (*P* = 0.0088, *n* = 8). **(j)** Escape latency in the five-testing days, significant differences were found between *3 M vs. 6 M (*P* = 0.0120, *n* = 8), **6 M vs. 12 M (*P* = 0.0464, *n* = 8), ^#^3 M vs. 12 M (*P* < 0.0001, *n* = 8), ^##^6 M vs. 12 M (*P* = 0.0001, *n* = 8), ^###^12 M vs. 18 M (*P* = 0.0010, *n* = 8), ^+^3 M vs. 12 M (*P* = 0.0006, *n* = 8) ^++^6 M vs. 12 M (*P* = 0.0122, *n* = 8), ^+++^12 M vs. 18 M (*P* = 0.0223, *n* = 8), ^&^3 M vs. 12 M (*P* = 0.0017, *n* = 8), and ^&&^6 M vs. 12 M groups(*P* = 0.0059, *n* = 8). **k)** Two-dimensional representation of the path of a representative mouse of each age during the memory phase. DI, Discrimination Index, *FAM* Familiarization phase of the NORT, *STM* Short-term memory phase of the NORT, *LTM* Long-term memory phase of the NORT, Novel Object Recognition Test, NORT

The results of the exploratory activity test in a novel environment show a significant (one way ANOVA, F (3, 36) = 21.99, *P* < 0.0001) and consistent decrease in locomotor activity with respect to age. Multiple comparison analysis shows that the decrease is significant among all groups except between 12 M and 18 M rodents (Fig. 2a. Tukey’s test, *P = 0.0077, **P = 0.0067, ***P < 0.0001, ^#^*P* = 0.0357, ^##^*P* = 0.0101). Figure 2e show two-dimensional representation of the path of a representative rodent of each age in the OFT.

#### Recognition memory

Analysis of data obtained from the NORT shows a significant decrease in ST (one way ANOVA, F (3, 36) = 3.469, *P* = 0.0293) and LT (one way ANOVA, F (3, 36) = 4.098, *P* = 0.0157) recognition memory. Multiple comparisons analysis shows that ST recognition memory was significantly decreased in 18 M rodents compared to the 6 M group (Fig. 2c. Tukey’s test, *P = 0.0385), while LT recognition memory was significantly reduced in 18 M rodents compared to 12 M (Fig. 2d. Tukey’s test, **P = 0.0107). Therefore, a constant trend towards a decline in this memory is observed, which becomes significant in the older groups. Two-dimensional representation of the path in the ST phase (f) and LT phase (g) of a representative rodent of each age in the NORT are showed in the Fig. 2. In addition to these analyses, our results shows that the total exploration time, i.e., the time spent exploring the novel object plus the time exploring de familiar object, is reduced in the 12 M and 18 M mice compared to the 3 M and the 6 M mice (see supplementary materials). This pattern is observed in the three phases of the NORT, but is more pronounced in the LT recognition memory phase.

#### Spatial memory

To determine the impact of age on spatial memory, the MWM was used. This test evaluated the rodent’s ability to remember the location of the escape platform. The results showed that 3 M mice took the least time to contact this platform for the first time, while 12 M and 18 M rodents took the longest. Statistical analysis showed significant differences where 12 M mice took significantly longer time compared to 3 M mice (Fig. 2h. one way ANOVA, F (3, 36) = 11.51, *P* = 0.0001; Tukey’s test, *P < 0.0001). Additionally, the amount of time each rodent spent in the escape quadrant was quantified. The results showed that 6 M mice spent the most time in the escape quadrant, while 12 M and 18 M rodents spent significantly less time (Fig. 2i. one way ANOVA, F (3, 36) = 5.301, *P* = 0.0051; Tukey’s test, *P = 0.0102, **P = 0.0088). Finally, the time taken by the mice to establish themselves on the escape platform was evaluated during the 5 days of testing (Fig. 2j). 6 M mice took significantly less time during the first-testing day compared to the 3 M and 12 M mice (one way ANOVA, F (3, 36) = 4.197, *P* = 0.0146; Tukey’s test, *P = 0.0120, **P = 0.0464). On the second-testing day, no significant differences were observed. On the third-testing day, it was observed that 12 M mice took significantly longer to locate themselves on the escape platform compared to 3 M, 6 M, and 18 M mice (one way ANOVA, F (3, 36) = 13.54, *P* < 0.0001; Tukey’s test, ^#^*P* < 0.0001, ^##^*P* = 0.0001, ^###^*P* = 0.0010). The same pattern was observed on the fourth-testing day (one way ANOVA, F (3, 36) = 7.496, *P* = 0.0008; Tukey’s test, ^+^*P* = 0.0006, ^++^*P* = 0.0122, ^+++^*P* = 0.0223). On the last-testing day, it was observed that 12 M mice took significantly longer than 3 M and 6 M mice to settle on the escape platform (one way ANOVA, F (3, 36) = 6.883, *P* = 0.0014; Tukey’s test, ^&^*P* = 0.0017, ^&&^*P* = 0.0059). Two-dimensional representation of the path of a representative mouse of each age during the memory phase in the MWM is presented in Fig. 2k.

### TDL and dendritic arborization are atrophied in NAcC and PFC, but not in CA1

#### Dendritic length

**Fig. 3:**
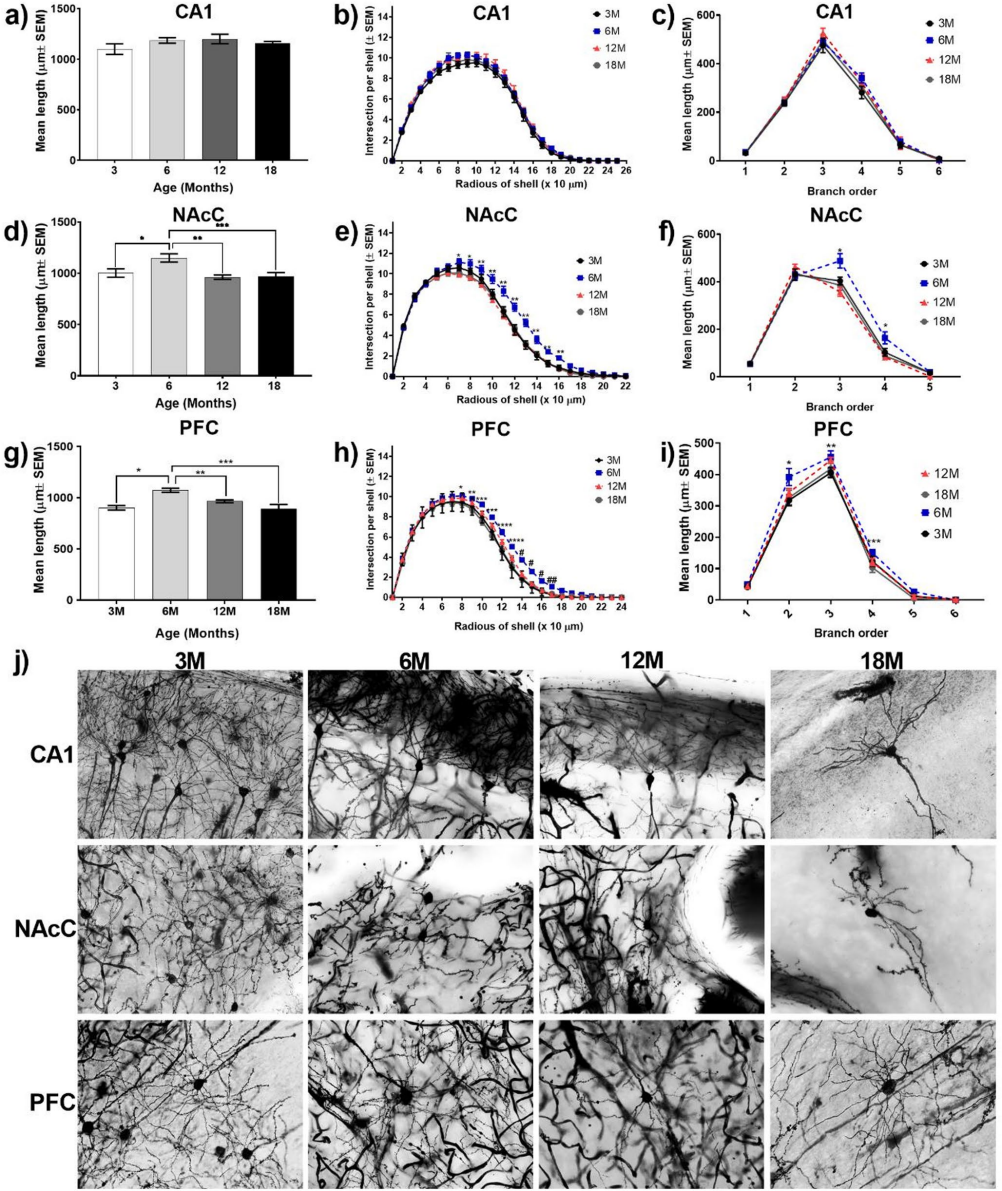
Dendritic arborization results in hippocampus, nucleus acumbens and prefrontal cortex. **(a)** Mean dendritic length by age group in CA1. **(b)** Dendritic arborization of CA1. **(c)** Dendritic length according to dendrite order of CA1. **(d)** Mean dendritic length by age group of NAcC, significant differences were found between 3 M vs. 6 M (*P < 0.0283, *n* = 10), **6 M vs. 12 M (*P* = 0.0035, *n* = 10) ***6 M vs. 18 M (*P* = 0.0061, *n* = 10). **(e)** Dendritic arborization of NAcC, significant differences were found between *6 M vs. 12M & 18 M (*n* = 10), **6 M vs. 3 M, 12M & 18 M(*n* = 10). **(f)** Dendritic length according to dendrite order of NAcC, significant differences were found between *6 M vs. 3 M, 12M & 18 M groups (*n* = 10). **(g)** Mean dendritic length by age group of PFC, significant differences were found between *3 M vs. 6 M (*P* = 0.0002, *n* = 10), **6 M vs. 12 M (*P* = 0.0274, *n* = 10), and ***6 M vs. 18 M groups (*P* = 0.0001, *n* = 10). **(h)** Dendritic arborization of PFC, significant differences were found between * 6 M vs. 18 M; ** 6 M vs. 3M & 18 M; *** 6 M vs. 3 M, 12M & 18 M: 12 M vs. 18 M; **** 6 M vs. 3 M, 12M & 18 M: 12 M vs. 3M & 18M; ^#^ 6 M vs. 3 M, 12M &18 M; and ^# #^ 6 M vs. 3M & 12 M groups (*n* = 10). **(i)** Dendritic length according to dendrite order of PFC, significant differences were found between *6 M vs. 3 M, 12M & 18 M; **6 M vs. 3 M; and ***6 M vs. 18 M groups (*n* = 10). **(j)** Representative photograph of CA1, NAcC and PFC neurons of each age

*CA1 hippocampus*. TDL was assessed with no significant differences found between any of the groups (Fig. 3a. one way ANOVA, F (3, 36) = 1.341, *P* = 0.2764). *NAcC*. An increase in dendritic length in 6 M mice compared to 3 M, 12 M and 18 M mice was observed, with no other differences between the other groups (Fig. 3d. one way ANOVA, F (3, 36) = 6.003, *P* = 0.0020; Tukey’s test, *P < 0.0283, **P = 0.0035, ***P = 0.0061). *PFC*. Pyramidal neurons of PFC show the same pattern as NAcC, with greater dendritic length observed in 6 M rodents compared to 3 M, 12 M, and 18 M rodents, with no other differences observed between the other groups (Fig. 3g. one way ANOVA, F (3, 36) = 10.31, *P* < 0.0001; Tukey’s test, *P = 0.0002, **P = 0.0274, ***P = 0.0001). Representative micrographs of CA1, NAcC, and PFC neurons at each age group (Fig. 3J).

#### Dendritic arborization

The dendritic morphology analysis in hippocampus shows no differences in the complexity of dendritic arborization, ergo, dendrites projections have a similar tridimensional bifurcation regarding to the distance with the soma (Fig. 3b. two way ANOVA, F (72, 900) = 0.4535, *P* > 0.9999). Same results were found in dendritic length according to dendrite order (Fig. 3c. two way ANOVA, F (15, 216) = 0.6824, *P* = 8005). In NAcC, a significative increase (Fig. 3e. two way ANOVA, F (69, 864) = 2.738, *P* < 0.0001; Tukey’s test *P* < 0.05, *6 M vs. 12M & 18 M; **6 M vs. 3 M, 12M & 18 M) in dendritic arborization complexity was found in 6 M mice in comparation with the 12 M and 18 M groups approximately between 80 and 160 μm from the soma. In the same way, the dendritic length was longer in the third and fourth order dendrites of 6 M mice in comparation with the other age groups (Fig. 3f. two way ANOVA, F (12, 174) = 3.774, *P* < 0.0001; Tukey’s test *P* < 0.05, *6 M vs. 3 M, 12M & 18 M). In pyramidal neurons of PFC, a similar pattern to that found in NAcC was observed. A significant increase (Fig. 3h. two way ANOVA, F (69, 864) = 2.835, *P* < 0.0001; Tukey’s test *P* < 0.05, * 6 M vs. 18 M; ** 6 M vs. 3M & 18 M; *** 6 M vs. 3 M, 12M & 18 M: 12 M vs. 18 M; **** 6 M vs. 3 M, 12M & 18 M: 12 M vs. 3M & 18 M; # 6 M vs. 3 M, 12M &18 M; # # 6 M vs. 3M & 12 M) in dendritic arborization in 6 M mice compared with the other groups was observed, this in dendrites present between 80 and 160 μm from the soma. On the other hand, second and third order dendrites show greater length in 6 M mice compared to the 3 M, 12 M and 18 M groups (Fig. 3i. two way ANOVA, F (15, 216) = 1.170, *P* = 0.2973; Tukey’s test *P* < 0.05, *6 M vs. 3 M, 12M & 18 M; **6 M vs. 3 M; ***6 M vs. 18 M).

### Dendritic spine density and morphology is altered in aged rodents

#### Dendritic spine density

**Fig. 4:**
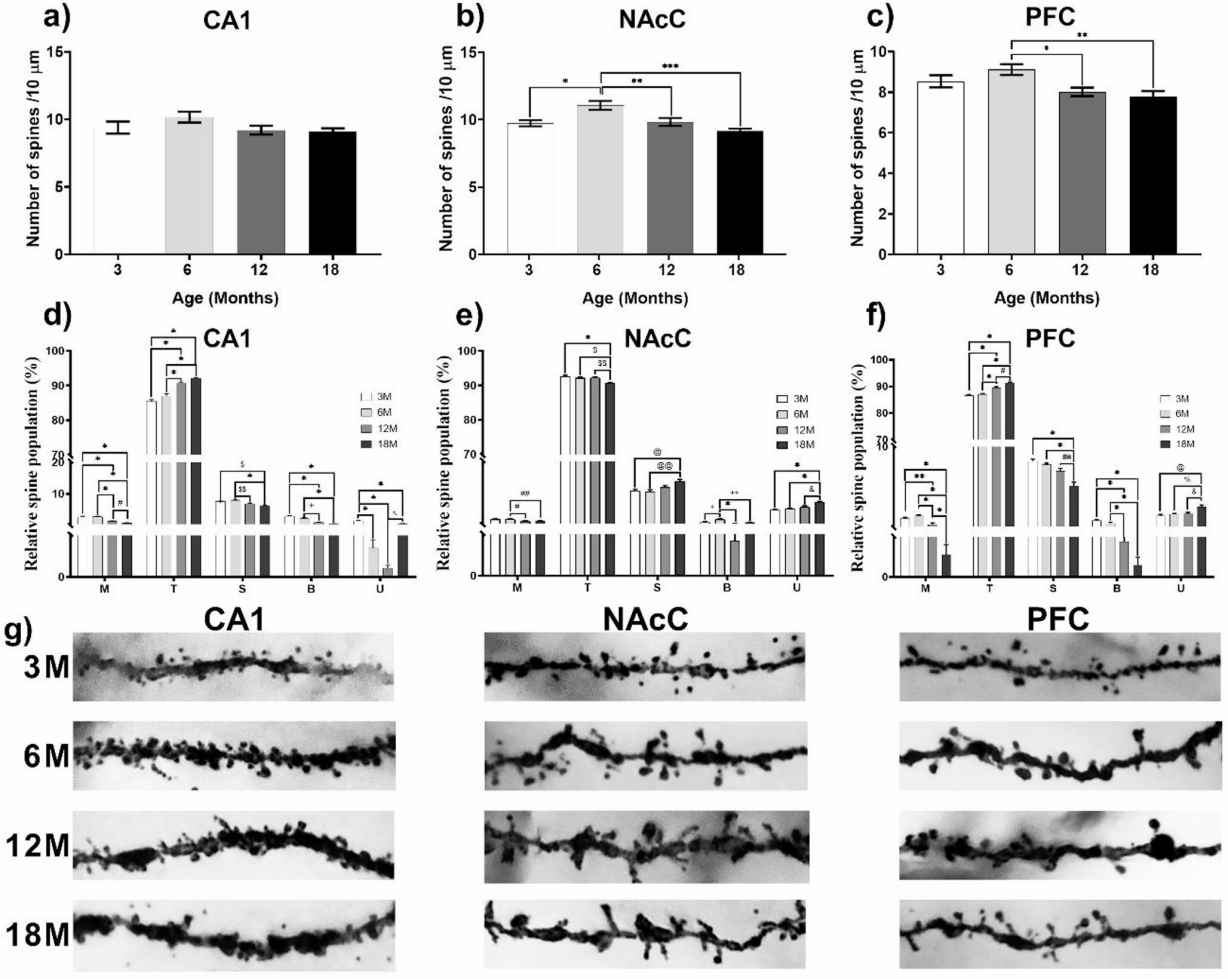
Changes in density and morphology of dendritic spines across age. **(a)** Dendritic spine density of CA1. **(b)** Dendritic spine density of NAcC, significant differences were found between *3 M vs. 6 M (*P* = 0.0080, *n* = 10), **6 M vs. 12 M (*P* = 0.0143, *n* = 10), and ***12 M vs. 18 M groups (*P* = 0.0001, *n* = 10). **(c)** Dendritic spine density of PFC, significant differences were found between *6 M vs. 12 M (*P* = 0.0267, *n* = 10) and **6 M vs. 18 M groups (*P* = 0.0051, *n* = 10). **(d)** Dendritic spine proportion according to morphology of CA1, (*P < 0.0001, ^#^*P* = 0.0192, ^$^*P* = 0.0011, ^$$^*P* = 0.0088, ^+^*P* = 0.0003, ^%^*P* = 0.0037; 3 M *n* = 9, 6 M *n* = 10, 12 M *n* = 10, & 18 M *n* = 10). **(e)** Dendritic spine proportion according to morphology of NAcC, (*P < 0.0001, #*P* = 0.0133, ##*P* = 0.0397, $*P* = 0.0011, $$*P* = 0.0006, @*P* = 0.0062, @@*P* = 0.0016, +*P* = 0.0121, ++*P* = 0.0044, &*P* = 0.0042; 3 M *n* = 8, 6 M *n* = 10, 12 M *n* = 10, & 18 M *n* = 10). **(f)** Dendritic spine proportion according to morphology of PFC, (*P < 0.0001, **: *P* = 0.0006, ^#^*P* = 0.0016, ^##^*P* = 0.0020, ^@^*P* = 0.0006, ^%^*P* = 0.0034, ^&^*P* = 0.0076; 3 M *n* = 10, 6 M *n* = 10, 12 M *n* = 10, & 18 M *n* = 10). **(g)** Representative photograph of CA1, NAcC and PFC dendritic spines of each age

*CA1 hippocampus*. Dendritic spine analysis showed no change (Fig. 4a. one way ANOVA, F (3, 36) = 1.801, *P* = 0.1644) in spine density between any of the groups. However, the analysis of dendritic spines according to their morphology shows significant changes in each type of dendritic spine (Fig. 4d). A significant age-induced decrease in mushroom spines was observed, mainly between 12 M and 18 M mice compared to 3 M and 6 M mice (one way ANOVA, F (3, 36) = 53.74, *P* < 0.0001). A significant age-related increase was shown in thin spines (one way ANOVA, F (3, 36) = 51.28, *P* < 0.0001). In stubby spines, a reduction was observed in 18 M mice compared to 3 M and 6 M mice, and 12 M mice had fewer stubby spines compared to the 6 M group (one way ANOVA, F (3, 36) = 11.29, *P* < 0.0001). In addition, a significant decrease was observed in bifurcated spines in 12 M and 18 M mice compared to 3 M and 6 M mice (one way ANOVA, F (3, 36) = 34.14, *P* < 0.0001). Regarding unclassified spines, a reduction was observed in 6 M, 12 M and 18 M rodents compared to 3 M mice, as well as a greater number in the 18 M group compared to the 12 M group (one way ANOVA, F (3, 36) = 53.21, *P* < 0.0001).

*NAcC*. Analysis showed a reduction in dendritic spines in 12 M and 18 M mice compared to 6 M mice, and an increase in dendritic spines in 6 M mice compared to 3 M mice (Fig. 4b. one way ANOVA, F (3, 36) = 8.578, *P* = 0.0002; Tukey’s test, *P = 0.0080, **P = 0.0143, ***P = 0.0001). Regarding dendric spines morphology, statistics analysis shows significant changes (Fig. 4e). 12 M and 18 M mice showed fewer mushroom spines compared to 6 M mice (one way ANOVA, F (3, 36) = 5.010, *P* = 0.0055). In thin spines, a reduction is also observed in the 18 M mice compared to the other ages (one way ANOVA, F (3, 36) = 11.23, *P* < 0.0001). On the contrary, stubby spines (one way ANOVA, F (3, 36) = 6.568, *P* = 0.0013) and unclassified spines (one way ANOVA, F (3, 36) = 12.87, *P* < 0.0001) show an increase in the 18 M rodents compared to those of 3 M and 6 M. Likewise, the 6 M rodents have a greater number of bifurcated spines (one way ANOVA, F (3, 36) = 9.450, *P* = 0.0001).

*PFC*. Quantification of dendritic spines shows a significant decrease in 12 M and 18 M mice compared to the 6 M group (Fig. 4c. one way ANOVA, F (3, 36) = 5.102, *P* = 0.0048; Tukey’s test, *P = 0.0267, **P = 0.0051). On the other hand, significant changes are observed in each type of dendritic spine (Fig. 4f). Regarding the number of mushroom spines, a significant decrease in the 12 M and 18 M groups compared to the 3 M and 6 M groups is observed, and the 18 M group has fewer spines than the 12 M group (one way ANOVA, F (3, 36) = 48.05, *P* < 0.0001). In thin spines, an increase is observed in the 12 M and 18 M groups compared to the 3 M and 6 M groups, even 18 M rodents have more thin spines than 12 M rodents (one way ANOVA, F (3, 36) = 47.03, *P* < 0.0001). In stubby spines, a tendency towards an age-related decrease is observed, where the 18 M group shows a significant reduction in stubby spines with respect to the younger rodents (one way ANOVA, F (3, 36) = 16.88, *P* < 0.0001). On the other hand, a significant reduction in bifurcated spines is observed in 12 M and 18 M mice compared to the 3 M and 6 M groups (one way ANOVA, F (3, 36) = 45.90, *P* < 0.0001). As for unclassified spines, these are increased in 18 M mice compared to the other groups (one way ANOVA, F (3, 36) = 7.725, *P* = 0.0004). Representative micrographs of dendritic spines in CA1, NAcC, and PFC across age groups are presented in Fig. 4g.

## Discussion

We assessed spatial and recognition memory, along with the quantitative analysis of dendritic morphology, including dendritic length, spine density, and spine type, at key developmental stages in mice. In the OFT, age-dependent reductions in locomotion were observed. Spatial memory showed reduced escape latency in 12 M mice compared to other groups. Furthermore, time spent in the target quadrant was decreased in both 12 M and 18 M. Recognition memory deficits were observed in animals at 18 M. Moreover, spine density analysis showed an increase at 6 M in the NAcC, whereas a reduction was noted at 12 M and 18 M in the PFC. Spine morphology analysis revealed age-dependent changes in complexity, with a notable increase in the proportion of thin spines in the CA1 and PFC regions and a corresponding reduction in the NAcC. No significant differences in dendritic arborization were found across the evaluated ages. However, TDL was increased at 6 M in the NAcC, and PFC compared to other groups.

### Age-dependent alterations in locomotion, recognition, and Spatial memory in mice

Cumulative evidence has shown that aged humans had spatial and recognition memory deficits (Caplan and Lipman 1995; Wilkniss et al. 1997; Moffat et al. 2001). It is of critical importance to evaluate two forms of memory and possible neuronal atrophy or rearrangement and modifications in spines in independent groups of mice representing key developmental stages. In this study, we evaluated these parameters across four independent groups of mice at 3 M, 6 M, 12 M and 18 M. Specifically, 3 M mice correspond to early adulthood, 6 M mice align with early middle age, 12 M mice represent midlife, and 18 M mice reflect the aging stage (Dutta and Sengupta 2016).

The MWM is possibly the most widely used test of spatial learning and memory in rodents (Morris 1984; Rosenzweig and Barnes 2003; Menard et al. 2015). We observed that while spatial learning was specially impaired in the 12 M group, spatial memory was evident in the 12 M and 18 M groups in this paradigm. In apparent agreement, progression of spatial learning slowed down at the 24 M and 32 M compared to the 12 M (Teglas et al. 2019). Moreover, Mota et al. (2019) compared young 4-6 M versus old 22-24 M rats and observed a reduced performance in old animals in the MWM. Therefore, these results in mice agree with previous findings observed in rats.

The NORT comprises a first day of habituation to the arena where locomotion is assessed in the task often referred as OFT. This test is a widely recognized to measure locomotion, where hypo- or hyperlocomotion are considered traits of various disorders (Ennaceur and Chazot 2016; Morales-Medina et al. 2017). In this study, we observed an age-related reduction in locomotion in this test in male mice. In apparent agreement, previous studies have observed a reduction in locomotion in rats as animals are aging (Salvatore et al. 2016; Teglas et al. 2019; Aguilar-Hernandez et al. 2020). Moreover, we observed a reduction in the DI in the 18 M group in the NORT. In male rats, DI increased during both retention phases in the 3 M and 6 M groups, whereas in the 18 M group, the increase was evident only in the short-term phase (Aguilar-Hernandez et al. 2020). Similarly, a reduction in the DI was reported in the 24 M and 32 M rats compared to the 12 M, suggesting deficits in recognition memory (Teglas et al. 2019). Aged (18-24 M) rats had similar exploration times in the long-term phase of object recognition, suggesting an impairment in the task compared to young male counterparts (3 M) (Arias-Cavieres et al. 2017). Therefore, recognition memory is affected by aging in mice and rats.

### Age-related modifications in dendrites and spines in the CA1 hippocampus, PFC, and NAcC in mice

Neurons are prone to modifications in the dendritic arbor and spines (number and type) due to internal or external stimuli including aging (Koleske 2013; Flores et al. 2020; von Bohlen Und Halbach 2023). Dendritic protrusions, known as filopodia, emerge and exhibit dynamic movement and are present in different shapes with specific characteristics (Flores et al. 2020). While the shape of the dendritic spines was described in the methodology, here we describe some functional properties (Yuste 2011; Harris and Weinberg 2012; Bello-Medina et al. 2016). Thin spines are prone to undergo structural changes in response to stimuli and upon stablishing contact with another neuron, these structures lose their motility. Mushroom spines are considered more stable and associated with strong, long-term connections. Stubby spines are measured as a transition between immature and mature spines. Bifurcated or branched spines may form multiple synapses and it is less common but important in complex synaptic integration.

Working and reference memory tasks both evaluate spatial learning and memory, but they operate on different temporal scales. Working memory relies on short-term retention and is critically dependent on hippocampal interactions with the PFC, whereas reference memory is associated with long-term retention and hippocampus-dependent (Morris 1984; Goldman-Rakic 1995; Kesner 2000). Therefore, it crucial for investigating age-related alterations in dendrites and spines in CA1 dorsal hippocampus. Structural changes in neuronal dendritic architecture have been shown to correlate with cognitive performance in both rodents and humans (Becker et al. 1986; Cerqueira et al. 2005; Menard et al. 2015). In the current study, although dendritic length and spine density remained largely unchanged across groups, we observed significant alterations in spine morphology within the CA1 hippocampus. Indeed, the 18 M group exhibited pronounced alterations in spine morphology, characterized by an age-dependent decrease in mushroom spines accompanied by an increase in thin spines; additionally stubby and bifurcated spines were also reduced in this aged group. In the context of aging, it was previously shown that memory impairments are not primarily due to neuronal loss, but rather alterations in dendritic morphology in rats (Burke and Barnes 2006; Dickstein et al. 2007). Indeed, our group observed a reduction in spine density in the 18 M group compared to 3 M and 6 M groups alongside a reduction in mushroom spines and an increase in stubby type of spines in the 18 M group in rats (Aguilar-Hernandez et al. 2020). Moreover, the apical dendritic tree is reduced in 22-24 M compared to 4-6 M rats in this region (Mota et al. 2019). Notably, in an electrophysiological experiment aged (18-24 M) presented a reduced field excitatory post-synaptic potential (fEPSP) slope, compared to young (3 M) rats. Finally, Rosenzweig and Barnes (2003) indicated a loss of functional synapses in the CA1 hippocampus. There are several pathways affected in aging. Among them, there is increased ryanodine receptor type 2 (RyR2) oxidation levels in aged rat that likely generate anomalous calcium signals, which may contribute to the well-known impairments in hippocampal LTP and spatial memory observed during aging (Arias-Cavieres et al. 2017). In the CA1, there is a decrease in cholinergic fiber density in the 24 M and 32 M groups compared to the 12 M group (Teglas et al. 2019). We observed an age-related effect of spine type in mice compared to major rearrangements shown in rats and this modifications in the type of spines could be responsible, at least partially, for the behavioral deficits observed in paradigms that measure different forms of memory in mice.

The PFC is implicated in numerous physiological processes including working memory, attention and more importantly integrating hippocampal information (Spellman et al. 2015; Joyce et al. 2025). Notably, the spine density is decreased in the cortex of aged humans (Benavides-Piccione et al. 2013). Moreover, the network PFC and hippocampus are integrated in memory processes, for example, during memory encoding, the hippocampus send information to the PFC and during retrieval, the PFC send information to the hippocampus (O’Neill et al. 2013; Spellman et al. 2015). In the present study, we observed major rearrangements in PFC neurons. There were decreases in dendritic length and spine density in the midlife and aging groups. Moreover, the 18 M group presented a dramatic decrease in mushroom spines. Previously, our group reported no differences in overall spine density among 3 M, 6 M and 18 M male rats, but observed a decrease in mushroom spines accompanied by an increase in stubby spines in the 18 M group (Aguilar-Hernandez et al. 2020). In contrast, dendritic complexity in mPFC neurons (layers II/III) of aged rats remained comparable to that of young animals (Mota et al. 2019). Moreover, numerous formulations have appeared in the market that improve cognitive processes during aging. For example, *Lycium barbarum* (Goji) increases neuronal arborization and density of dendritic spines in various brain regions including the PFC and dorsal hippocampus in18M rats (Ruiz-Salinas et al. 2020).

The NAcC is part of the basal ganglia involved in the integration of spatial and reward-related information (Cauli et al. 2005; Braun et al. 2016). In the present study, we observed a decrease in spine density in the middle and aged groups along a decrease in mushroom and thin spines. There was an increase in stubby, bifurcated and unclassified spines. Moreover, locomotion has been associated with modifications in dopaminergic transmission. For example, aged rats present a reduced levels of tyrosine hydroxylase and dopamine in the NAcC and caudate-putamen compared to young counterparts (Mora et al. 2008; Tomm et al. 2018). Therefore, modifications in the number and type of spines could be implicated in the age-induced reduction in locomotion. Moreover, the age-dependent alterations observed in dendrites and spines in the PFC-hippocampus-NAcC could contribute to the alterations in spatial and recognition memory.

A limitation of the present study is the exclusive use of male rats, which precludes the assessment of potential sex-specific differences in neuronal morphology across critical developmental stages. Given the growing evidence of sex-dependent trajectories in brain maturation and plasticity, the absence of female subjects limits the generalizability of the findings and highlights the need for future studies to incorporate both sexes.

## Conclusion

Aging is associated with deficits in learning and memory, hippocampal damage and alterations to the NAcC-PFC-hippocampal network. Most importantly, we observed numerous modifications region-specific in the type of spines during aging. These results emphasize the potential for anti-aging interventions targeting synaptic structures to extend the healthspan of aging individuals.

## Supplementary Information

Below is the link to the electronic supplementary material.

Supplementary Material 1

## Data Availability

Data supporting the findings of this study are available from the corresponding author upon reasonable request.
