# Supplementary material for: Dendritic spine degeneration is associated with age-related decline in recognition and spatial memory in male mice
Source: Brain Struct Funct. 2025 Aug 28;230(7):142. doi: 10.1007/s00429-025-03002-7 (PMC12394372; doi:10.1007/s00429-025-03002-7)
Supplement: Supplementary file 1 — Supplementary Material 1 [file 429_2025_3002_MOESM1_ESM.docx]

**Supplementary material**

**Table 1**. Results of the NORT. The three phases of the NORT are shown: familiarization, short-term memory, and long-term memory. The corresponding section presents the mean time in second that each group spent interacting with the familiar or novel object. The standard error of the mean and the standard deviation are included.

| Time (s) of interaction with the objects in the NORT | | | | | | | | | |
| --- | --- | --- | --- | --- | --- | --- | --- | --- | --- |
|  |  | **3M** | | **6M** | | **12M** | | **18M** | |
|  |  | **Familiar object** | **Novel object** | **Familiar object** | **Novel object** | **Familiar object** | **Novel object** | **Familiar object** | **Novel object** |
| Fam | **Mean** | 12.37 | 12.425 | 14.075 | 13.525 | 5.7 | 5.713 | 7.925 | 8.063 |
|  | **SD** | 8.373 | 7.738 | 1.926 | 3.311 | 1.542 | 1.536 | 5.045 | 3.683 |
|  | **SEM** | 2.960 | 2.736 | 0.681 | 1.171 | 0.545 | 0.542 | 1.784 | 1.302 |
| STM | **Mean** | 8.7 | 22.325 | 6.3875 | 19.4875 | 3.4125 | 7.65 | 8.9 | 9.625 |
|  | **SD** | 4.258 | 10.874 | 3.104 | 8.036 | 1.937 | 4.763 | 6.692 | 7.203 |
|  | **SEM** | 1.505 | 3.844 | 1.097 | 2.841 | 0.685 | 1.684 | 2.366 | 2.547 |
| LTM | **Mean** | 6.85 | 12.775 | 6.563 | 11.35 | 1.813 | 4.713 | 4.913 | 5.025 |
|  | **SD** | 3.182 | 4.954 | 2.289 | 4.840 | 0.993 | 2.991 | 2.530 | 2.414 |
|  | **SEM** | 1.125 | 1.752 | 0.809 | 1.711 | 0.351 | 1.057 | 0.894 | 0.853 |

NORT: Novel object recognition test, FAM: Familiarization phase, STM: Short-term memory phase, LTM: Long-term memory phase, SD: Standard deviation, SEM: Standard error of the mean, S: Seconds


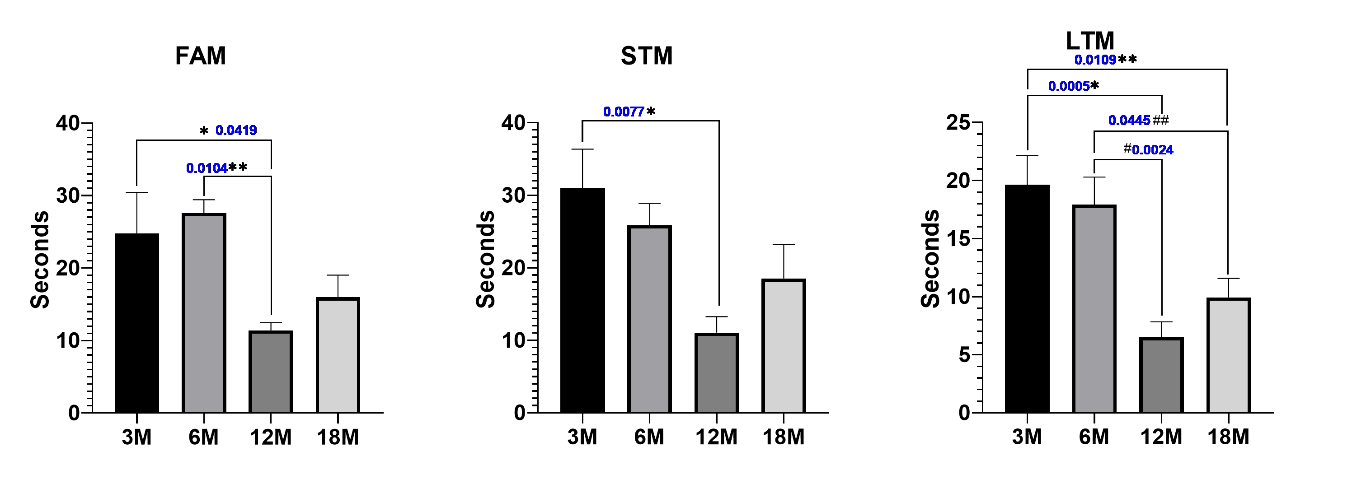


**Supplementary figure**: Total time exploration in NORT. The mean of total seconds spends exploring is showed in the three phases of NORT. The mice of 12M showed a significant decrease in the total time exploration in comparison with the 3M and 6M mice in the three phases. The 18M mice show a reduced total time exploration in contrast with the 3M and 6M mice only in the LTM phase.

*FAM: One way ANOVA, F (3, 28) = 5.014, P = 0.0066; Tukey’s test, *P = 0.0419, **P = 0.0104.*

*STM: One way ANOVA, F (3, 28) = 4.717, P = 0.0087; Tukey’s test, *P = 0.0077.*

*LTM: One way ANOVA, F (3, 28) = 9.605, P = 0.0002; Tukey’s test, *P = 0.0005, **P = 0.0109, ^#^P = 0.0024, ^##^P = 0.0445.*
